# The chromosome-level genome sequences of the freshwater sponge, *Spongilla lacustris *(Linnaeus, 1759) and the chlorophyte cobiont *Choricystis* sp., and the associated microbial metagenome sequences

**DOI:** 10.12688/wellcomeopenres.23988.1

**Published:** 2025-04-25

**Authors:** Sally P. Leys, Ute Hentschel, Graeme Oatley, Elizabeth Sinclair, Eerik Aunin, Noah Gettle, Camilla Santos, Michael Paulini, Dirk Erpenbeck, Haoyu Niu, Victoria McKenna, Wellcome Sanger Institute Tree of Life Management, Samples and Laboratory Team, Wellcome Sanger Institute Scientific Operations: Sequencing Operations, Wellcome Sanger Institute Tree of Life Core Informatics Team, EBI Aquatic Symbiosis Genomics Data Portal Team, Aquatic Symbiosis Genomics Project Leadership

**Affiliations:** 1 University of Alberta Department of Biological Sciences, Edmonton, Alberta, Canada; 2 RU Marine Symbioses, Helmholtz Centre for Ocean Research, Kiel, Schleswig-Holstein, Germany; 3 Tree of Life, Wellcome Sanger Institute, Hinxton, England, UK

**Keywords:** Spongilla lacustris, freshwater sponge, genome sequence, chromosomal, Spongillida; microbial metagenome assembly

## Abstract

We present a genome assembly from an individual *Spongilla lacustris* (freshwater sponge; Porifera; Demospongiae; Spongillida; Spongillidae). The genome sequence is 248.7 megabases in span. Most of the assembly is scaffolded into 23 chromosomal pseudomolecules. The mitochondrial genome has also been assembled and is 28.04 kilobases in length. A 14.6-megabase genome assembly of the green algal cobiont *Choricystis* sp. (Chlorophyta; Trebouxiophyceae) was scaffolded into 16 chromosomal pseudomolecules. Additionally, three bacterial metagenome bins were recovered from the same sample. Gene annotation of this assembly at Ensembl identified 30,435 protein coding genes.

## Species taxonomy: host

Eukaryota; Opisthokonta; Metazoa; Porifera; Demospongiae; Heteroscleromorpha; Spongillida; Spongillidae; *Spongilla*; *Spongilla lacustris* (Linnaeus, 1759) (NCBI:txid6055).

## Species taxonomy: eukaryote cobiont

Eukaryota; Viridiplantae; Chlorophyta; core chlorophytes; Trebouxiophyceae; Trebouxiophyceae *incertae sedis*; *Choricystis* clade; *Choricystis*; unclassified *Choricystis* (NCBI:txid3025909).

## Background

**Figure 1.**
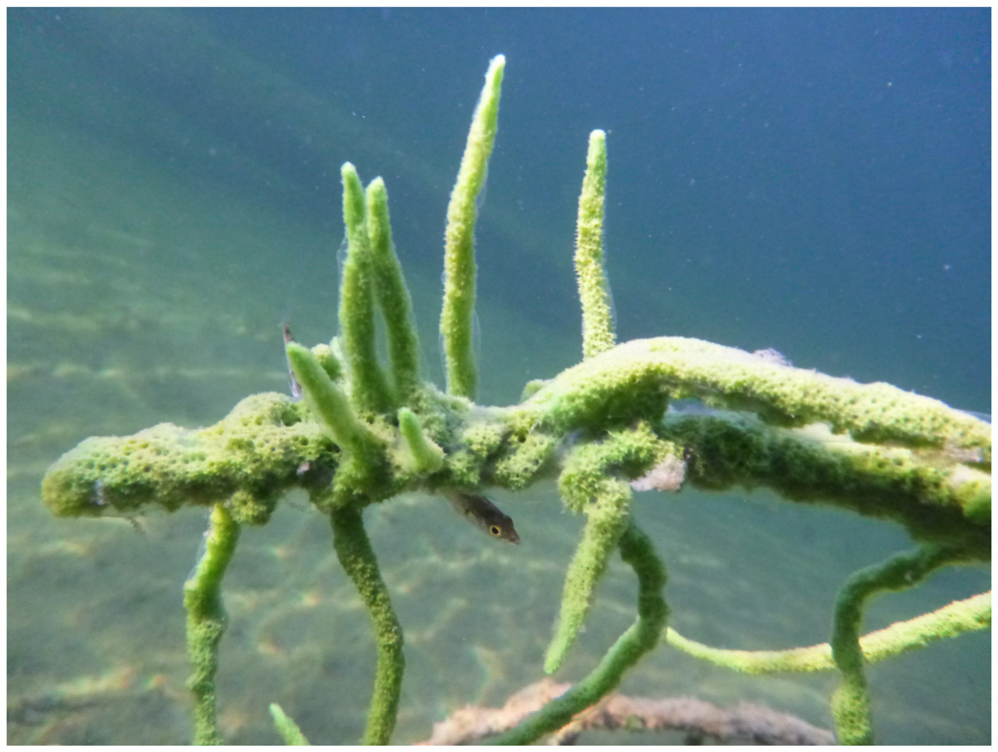
Image of a representative *Spongilla lacustris* (odSpoLacu1) specimen, similar to the one used for genome sequencing. Photograph by Sally Leys.

*Spongilla lacustris* (Linnaeus 1758) is a freshwater sponge with a Holarctic distribution ( Manconi & Pronzato, 2008). It is one of the most commonly encountered sponges in shallow 0–4 m depths in lakes and rivers of Europe and North America, where it thinly encrusts logs, sticks, rock as well as man-made structures such as the edges of bridges and canals. The colour is usually olive to bright green; when protected from light, it is yellow. A distinguishing feature of *S. lacustris* is the finger-like projections that can rise tens of centimetres up into the water (see Figure 1).

*S. lacustris* is uniform in cross section, with choanocyte chambers distributed across the full depth of the 1 cm to 2.5 cm-thick crust. The skeleton is siliceous, with megascleres of smooth, slightly curved oxeas and microscleres of spined, slightly curved oxeas ( Evans & Montagnes, 2019; Manconi & Pronzato, 2008). Spicules are incorporated into the overwintering cyst (the gemmule); these spicules, called gemmoscleres, are slightly spined oxeas. Gemmules vary greatly in the number of gemmoscleres they have, with some densely covered and others lacking spicules altogether.

Reproduction is both sexual and asexual. Freshwater sponges are gonochoristic and viviparous, with individuals producing sperm or eggs in mid-summer, brooding embryos through their development, and releasing them as larvae in July or early August ( Saller & Weissenfels, 1985). Larvae differ from those of other sponges in having adult characters such as choanocyte chambers and epithelial-lined canals as well as a large epithelial-lined cavity; at metamorphosis some of these features are retained and incorporated into the sponge’s aquiferous system. The end of summer triggers the formation of asexually produced gemmules into which the entire sponge is packaged as dedifferentiated cells called thesocytes. Thesocytes undergo karyokinesis and exist as binucleate cells until the end of dormancy in early spring ( Harrison *et al.*, 1981). Hatching is associated with a rise in cyclic GMP and cyclic AMP which are thought to be involved in mobilisation of nutrients and in ion transport that takes place during hatching and early development ( Harrison *et al.*, 1981).

Endosymbiosis in freshwater sponges has been studied for more than a century ( Brandt, 1881). Algae isolated from *Spongilla lacustris* individuals consist of different coccoid species, some hosting *Chlorella sorokiniana*/vulgaris and others *Choricystis parasitica* ( Huss *et al.*, 1986; Lewin, 1966) and a *Chlorella*-like alga, *Lewiniosphaera symbiontica* ( Pröschold & Darienko, 2020). White individuals are typically aposymbiotic ( Williamson, 1979). Algal symbionts are taken up from water during filtration and passed from choanocytes to other cell types ( Saller, 1991). Physiological studies have found that although the algal symbionts of *S. lacustris* fixed more carbon than did algae in Hydra and Paramecium, they provided very little product to the host and only in the form of glucose rather than maltose ( Muscatine *et al.*, 1967). Nevertheless, despite this presumably low energy transfer from the algae, sponges with algal symbionts hatch faster and grow better than those without ( Frost & Williamson, 1980; Saller, 1991). While only about half of *Spongilla lacustris* gemmules have photosynthetic algal symbionts ( Frost & Williamson, 1980), it has been suggested that in harsh years, hatching of aposymbiotic gemmules later than those with algal symbionts might ensure survival of the population ( Frost & Williamson, 1980).

The microbial diversity of *S. lacustris* was found to be limited in comparison to that of marine sponges, resembling that of the surrounding lake water ( Gernert *et al.*, 2005). Recent work combining microbial cultivation, metagenomics and natural product chemistry revealed a high potential for secondary metabolite production ( Graffius *et al.*, 2023). Ultrastructural studies have shown that the mesohyl of *S. lacustris* is essentially symbiont free ( Gernert *et al.*, 2005; Williamson, 1979). Bacteria were not found in any cell of *S. lacustris,* but digested bacterial pieces were found in archaeocytes ( Gernert *et al.*, 2005). In contrast, algae are found intracellularly in choanocytes and in large numbers in archaeocytes in green individuals of *S. lacustris,* but are rare in white individuals.

The wide distribution of *Spongilla lacustris* and its easy access in rivers and lakes have led to a broad range of studies on its cell biology ( Fjerdingstad, 1961; Musser *et al.*, 2021; Wachtmann *et al.*, 1990; Weissenfels *et al.*, 1990), physiology and development ( Adams, 2010; Brauer, 1975; Frost, 1980; Imsiecke, 1993; Saller, 1988; Saller & Weissenfels, 1985), as well as symbioses ( Brøndsted & Brøndsted, 1953; Francis *et al.*, 2016; Gernert *et al.*, 2005; Graffius *et al.*, 2023; Harrison *et al.*, 1981; Pröschold & Darienko, 2020). Such a breadth of knowledge on one species makes this an excellent model system for a diversity of future work.

## Genome sequence report for the host and chlorophyte cobiont

The genome was sequenced from a specimen of *Spongilla lacustris* grown in the laboratory at the University of Alberta, Edmonton, Canada (latitude 53.53, longitude –113.53). For the host organism, an estimated 34x coverage in Pacific Biosciences single-molecule HiFi long reads was generated. For the *Choricystis* sp. cobiont, 16× coverage was obtained, based on the estimated genome size. In both cases, the primary assembly contigs were scaffolded with chromosome conformation Hi-C data. Manual assembly curation corrected 239 missing joins or mis-joins and removed 160 haplotypic duplications, reducing the assembly length by 3.50% and the scaffold number by 77.02%, and increasing the scaffold N50 by 18.29%.

The final *Spongilla lacustris* assembly has a total length of 248.7 Mb in 70 sequence scaffolds with a scaffold N50 of 10.1 Mb ( Table 1). Most (99.73%) of the assembly sequence was assigned to 23 chromosomal-level scaffolds. During curation, we noted that chromosome 7 of the host assembly contains a collapsed repeat at 3.21 Mb that joins the chromosome arms. The final *Choricystis* sp. assembly has a total length of 14.5 Mb in 17 sequence scaffolds with a scaffold N50 of 1.0 Mb ( Table 1).

**Table 1.** Genome data for *Spongilla lacustris*, odSpoLacu1.1.

Project accession data		
Assembly identifier	odSpoLacu1.1	
Species	*Spongilla lacustris*	
Specimen	odSpoLacu1	
NCBI taxonomy ID	6055	
BioProject	PRJEB58940	
BioSample ID	SAMEA8580182	
Isolate information	odSpoLacu1	
Raw data accessions		
PacificBiosciences SEQUEL II	ERR10802379, ERR10798424, ERR10802378	
Hi-C Illumina	ERR10786015, ERR10786016, ERR10786017	
PolyA RNA-Seq Illumina	ERR12708738	
Assembly metrics		
Genome assembly	odSpoLacu1.1	ucChoSpea1.1
Assembly accession	GCA_949361645.1 (alternate haplotype: GCA_949361655.2)	GCA_958009055.1 (alternate haplotype: GCA_958009065.1)
Percentage of primary assembly mapped to chromosomes	99.73%	98.95%
Span (Mb)	248.7	14.5
Number of contigs	1,495	30
Contig N50 length (Mb)	0.3	0.7
Number of scaffolds	70	17
Scaffold N50 length (Mb)	10.1	1.0
Longest scaffold (Mb)	31.14	1.52
Consensus quality (QV)	53.6	51.1
BUSCO	C:64.4%[S:63.9%,D:0.4%], F:9.4%,M:26.2%,n:954	C:88.4%[S:88.0%,D:0.5%], F:1.6%,M:10.0%,n:1,519
Organelles	Mitochondrial genome: 28.04 kb	Mitochondrial genome: 34.74 kb; Plastid genome: 91.10 kb
Genome annotation of *Spongilla lacustris* GCA_949361645.1		
Number of protein-coding genes	30,435	
Number of non-coding genes	854	
Number of gene transcripts	43,627	

* BUSCO scores based on the metazoa_odb10 BUSCO set using version 5.3.2. C = complete [S = single copy, D = duplicated], F = fragmented, M = missing, n = number of orthologues in comparison. A full set of BUSCO scores is available at https://blobtoolkit.genomehubs.org/view/CASHZP01/dataset/CASHZP01/busco.

**Figure 2.**
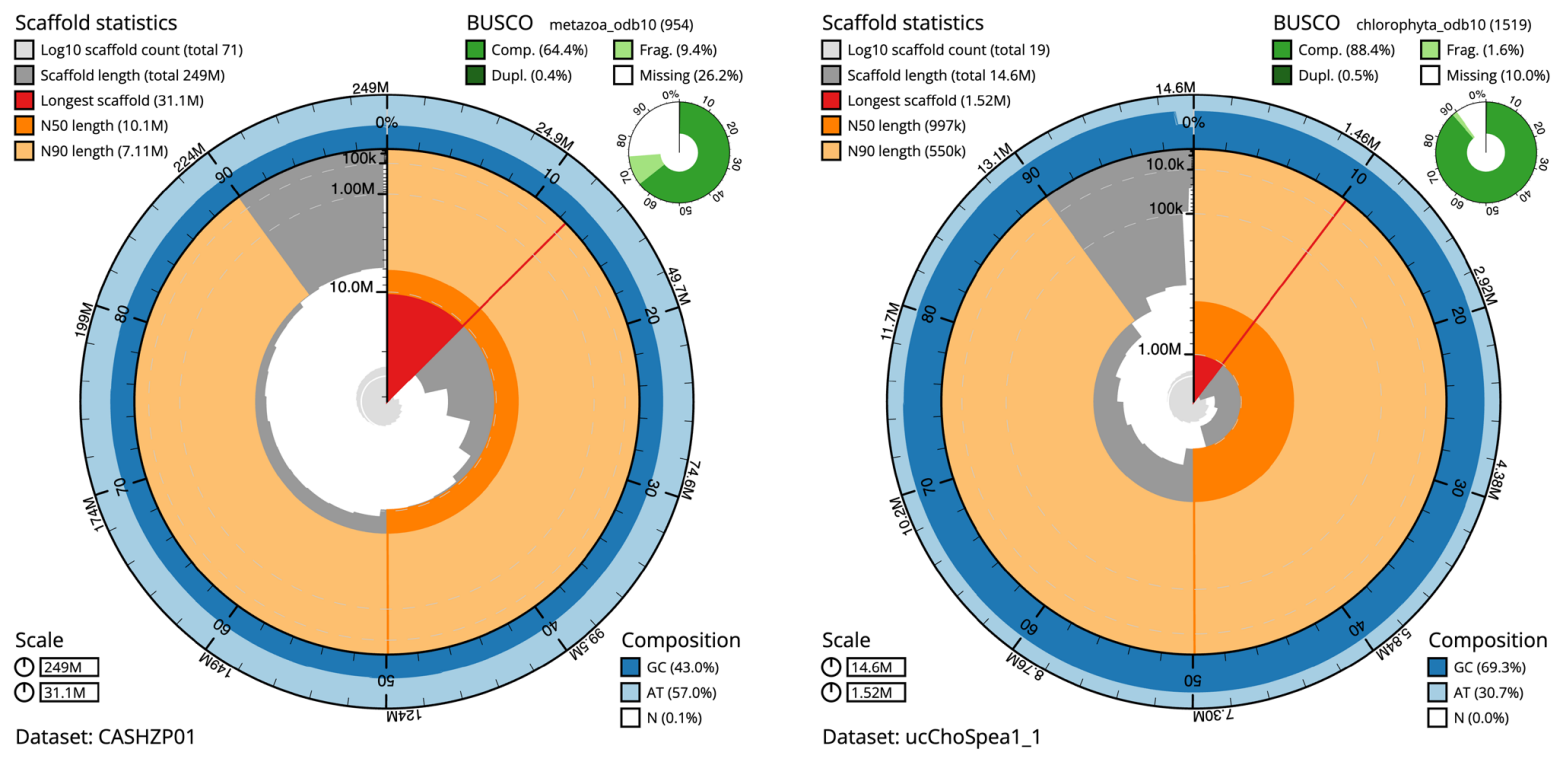
**A**. Snail plot summary of assembly statistics for assembly odSpoLacu1.1: An interactive version of this figure is available here. **B**. Snail plot summary of assembly statistics for assembly ucChoSpea1.1: An interactive version of this figure is available here. The BlobToolKit snail plots show N50 metrics and BUSCO gene completeness. The main plot is divided into 1,000 bins around the circumference with each bin representing 0.1% of the assembly. The distribution of scaffold lengths is shown in dark grey with the plot radius scaled to the longest scaffold present in the assembly (shown in red). Orange and pale-orange arcs show the N50 and N90 scaffold lengths respectively. The pale grey spiral shows the cumulative scaffold count on a log scale with white scale lines showing successive orders of magnitude. The blue and pale-blue area around the outside of the plot shows the distribution of GC, AT and N percentages in the same bins as the inner plot. A summary of complete, fragmented, duplicated and missing BUSCO genes is shown in the top right.

**Figure 3.**
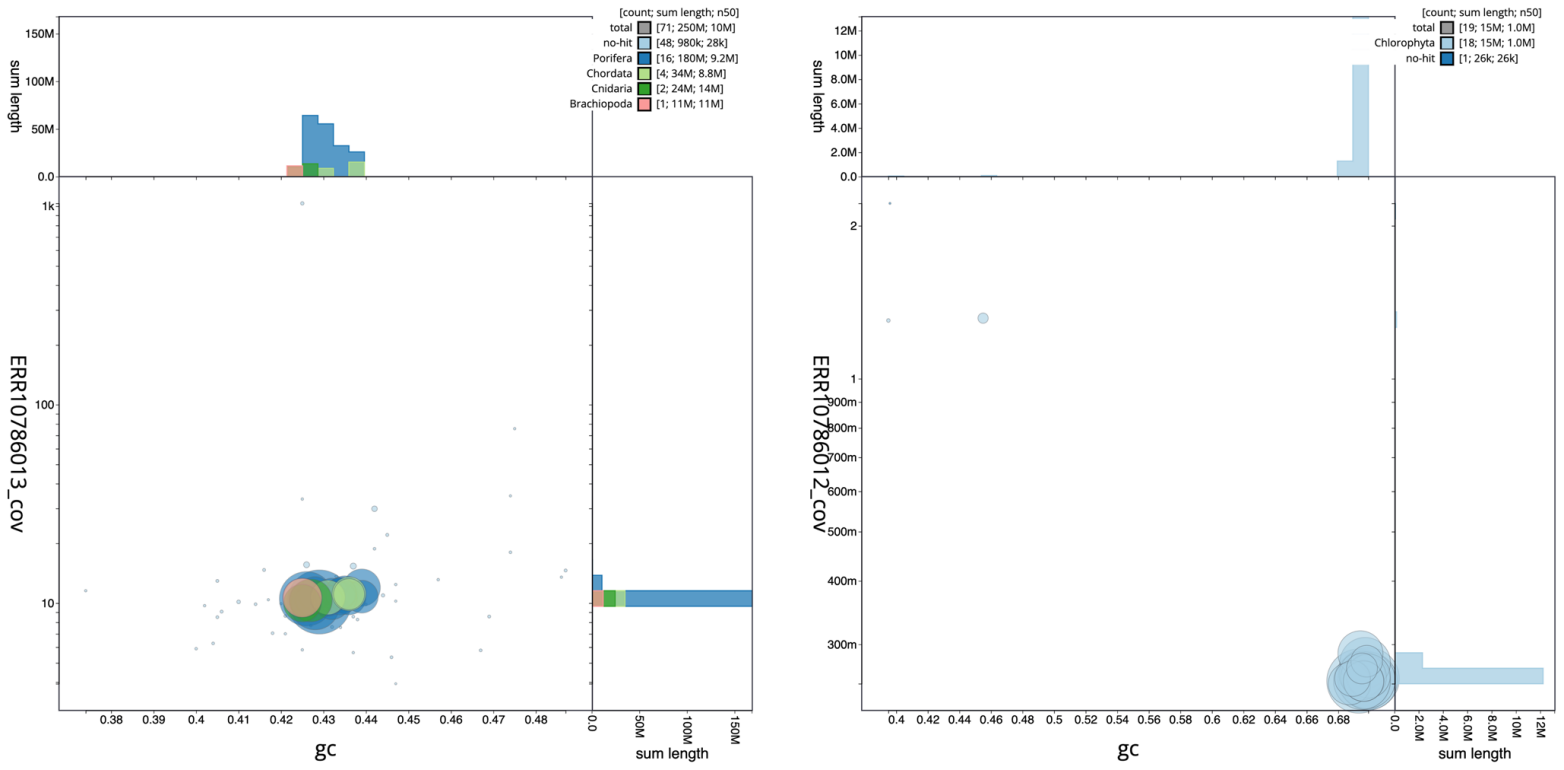
BlobToolKit GC-coverage plots. Scaffolds are coloured by phylum. Circles are sized in proportion to scaffold length. Histograms show the distribution of scaffold length sum along each axis. **A**. Genome assembly of *Spongilla lacustris*, odSpoLacu1.1: An interactive version of this figure is available here. **B**. Genome assembly of *Choricystis sp. odSpoLacu1*, ucChoSpea1.1: An interactive version of this figure is available here.

**Figure 4.**
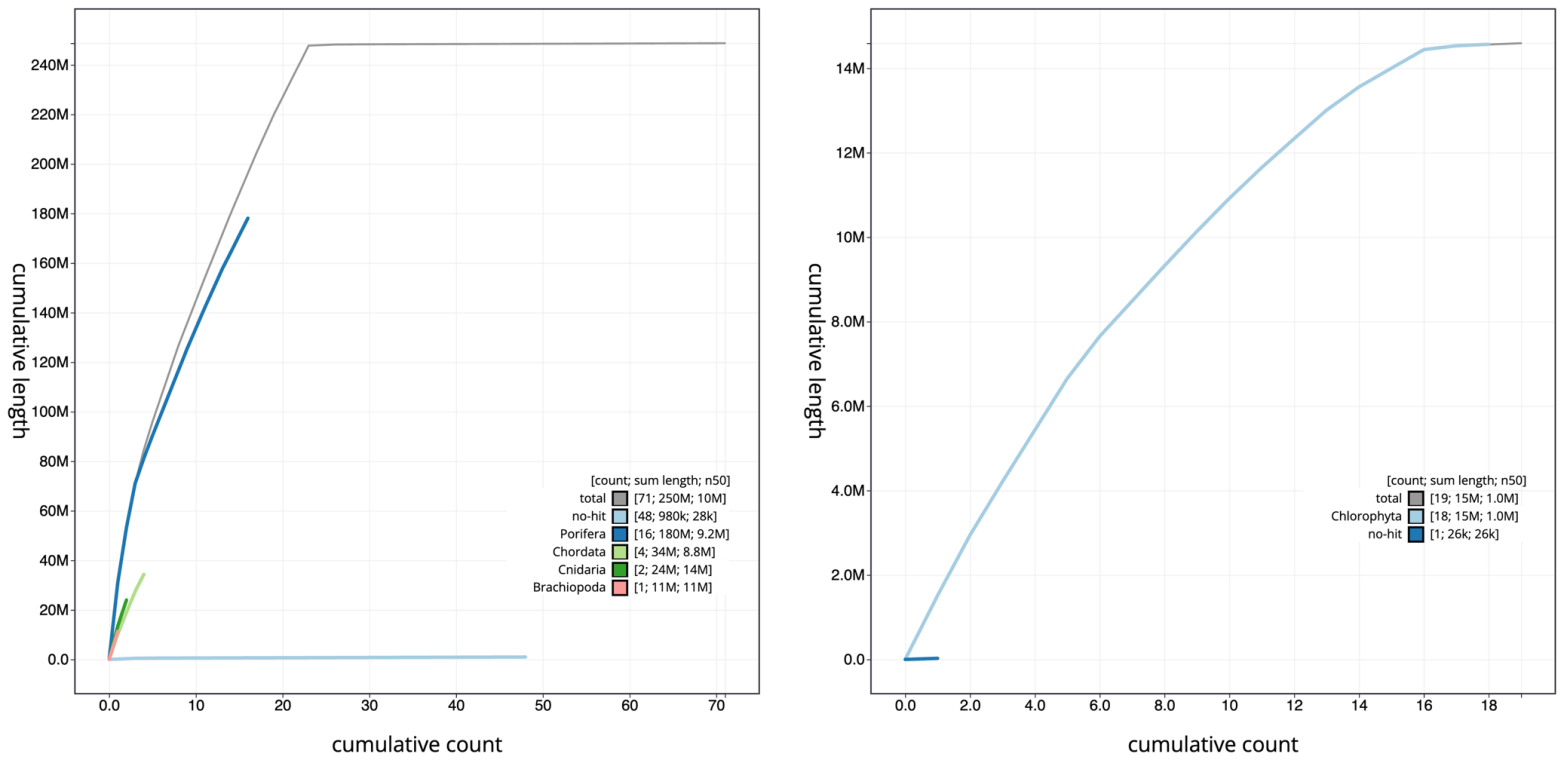
BlobToolKit cumulative sequence plots. The grey line shows cumulative length for all scaffolds. Coloured lines show cumulative lengths of scaffolds assigned to each phylum using the buscogenes taxrule. **A**. Cumulative sequence length for assembly odSpoLacu1.1: An interactive version of this figure is available here. **B**. Cumulative sequence length for assembly ucChoSpea1.1: An interactive version of this figure is available here.

**Figure 5.**
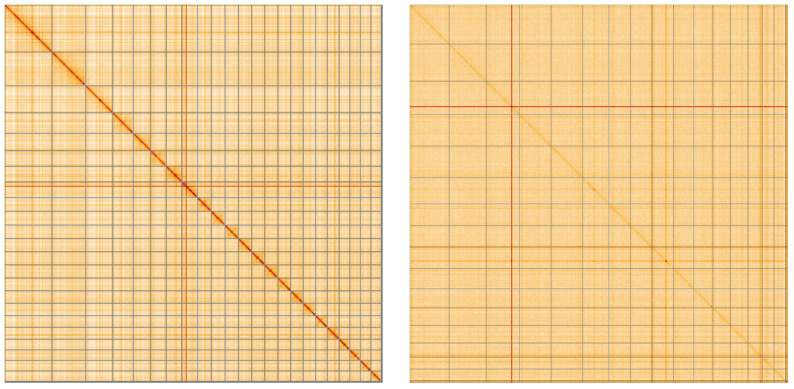
Hi-C contact maps visualised using HiGlass. Chromosomes are shown in order of size from left to right and top to bottom. **A**. Genome assembly of *Spongilla lacustris*, odSpoLacu1.1: An interactive version of this figure may be viewed here. **B**. Genome assembly of *Choricystis* sp., ucChoSpea1.1: An interactive version of this figure may be viewed here.

The snail plots in Figure 2 summarise the host and chlorophyte cobiont assembly statistics, while the distribution of assembly scaffolds on GC proportion and coverage for each assembly is shown in Figure 3. The cumulative assembly plots in Figure 4 show curves for subsets of scaffolds assigned to different phyla. Chromosome-scale scaffolds confirmed by the Hi-C data for both eukaryote assemblies are named in order of size ( Figure 5; Table 2 and Table 3).

**Table 2.** Chromosomal pseudomolecules in the genome assembly of *Spongilla lacustris*, odSpoLacu1.

INSDC accession	Chromosome	Length (Mb)	GC%
OX442415.1	1	31.14	43.0
OX442416.1	2	22.15	42.5
OX442417.1	3	17.78	43.0
OX442418.1	4	13.56	42.5
OX442419.1	5	11.34	42.5
OX442420.1	6	10.42	42.5
OX442421.1	7	10.12	44.0
OX442422.1	8	10.27	42.5
OX442423.1	9	9.18	42.5
OX442424.1	10	8.96	43.5
OX442425.1	11	8.87	43.0
OX442426.1	12	8.76	43.0
OX442427.1	13	8.68	43.5
OX442428.1	14	8.64	43.0
OX442429.1	15	8.4	43.5
OX442430.1	16	8.2	43.0
OX442431.1	17	8.08	43.5
OX442432.1	18	7.77	44.0
OX442433.1	19	7.72	43.5
OX442434.1	20	7.11	43.5
OX442435.1	21	6.92	42.5
OX442436.1	22	6.92	43.5
OX442437.1	23	6.79	43.0
OX442438.1	MT	0.03	43.0

**Table 3.** Chromosomal pseudomolecules in the genome assembly of *Choricystis sp. odSpoLacu1*, ucChoSpea1.

INSDC accession	Chromosome	Length (Mb)	GC%
OY253649.1	1	1.52	69.5
OY253650.1	2	1.43	70.0
OY253651.1	3	1.27	70.0
OY253652.1	4	1.23	70.0
OY253653.1	5	1.22	69.5
OY253654.1	6	1.0	69.5
OY253655.1	7	0.84	69.5
OY253656.1	8	0.83	69.5
OY253657.1	9	0.82	69.5
OY253658.1	10	0.78	70.0
OY253659.1	11	0.73	68.5
OY253661.1	13	0.68	69.5
OY253660.1	12	0.68	69.5
OY253662.1	14	0.55	69.0
OY253663.1	15	0.45	70.0
OY253664.1	16	0.43	69.5
OY253666.1	Pltd	0.09	45.5
OY253665.1	MT	0.03	39.5

The estimated Quality Value (QV) for the primary and alternate haplotype of the host assembly are both 53.6, and for the chlorophyte cobiont 51.1. The *Spongilla lacustris* assembly has a BUSCO v5.3.2 completeness of 64.4% (single = 63.9%, duplicated = 0.4%), using the metazoa_odb10 reference set ( *n* = 954). The *Choricystis* sp. genome shows a BUSCO completeness of 88.4% (single: 88.0%, duplicated: 0.5%) using the chlorophyta_odb10 reference set ( *n* = 1,519).

## Metagenome report

**Figure 6.**
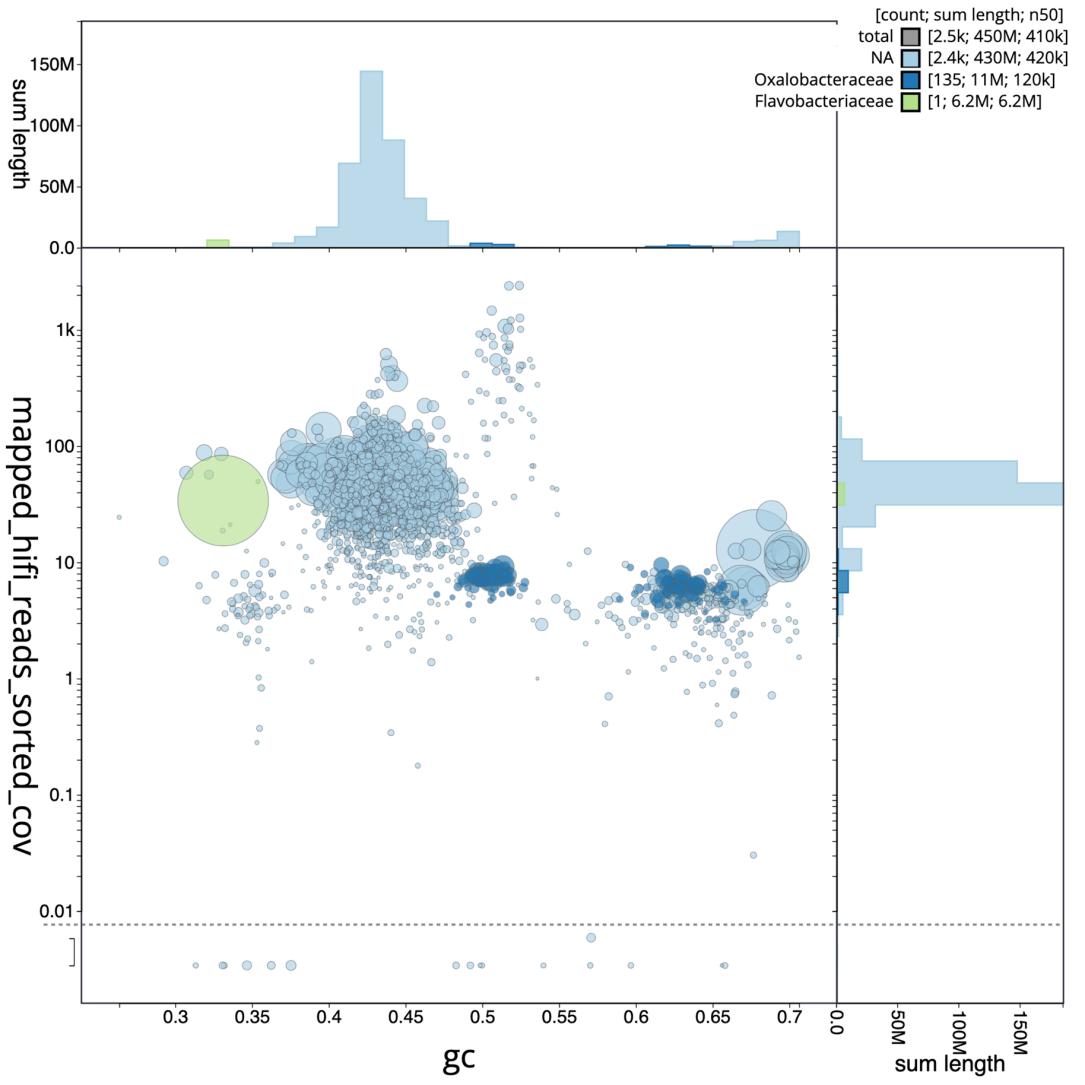
Blob plot of base coverage in mapped against GC proportion for sequences in the *Spongilla lacustris* metagenome. Binned contigs are coloured by family. Circles are sized in proportion to sequence length on a square-root scale, ranging from 2,309 to 6,234,830. Histograms show the distribution of sequence length sum along each axis. An interactive version of this figure may be viewed here.

Three binned genomes were generated from the metagenome assembly ( Figure 6) of which one was classified as a high quality metagenome assembled genome (MAG) (see methods) The completeness values for these assemblies range from approximately 79% to 99%, with contamination below 3%. For details on binned genomes see Table 4.

**Table 4.** Quality metrics and taxonomic assignments of the metagenome bins.

Quality	Size (bp)	Contigs	Completeness	Contamination	Circular	Taxon	NCBI taxid
Medium	4,838,303	68	79.23%	0.13%	N	uncultured *Pseudoduganella* sp.	1663410
Medium	6,800,618	567	89.44%	2.18%	N	uncultured *Undibacterium* sp.	686278
High	6,359,527	1	99.65%	1.39%	N	uncultured *Flavobacterium* sp.	165435

## Genome annotation report

The *Spongilla lacustris* genome was annotated by Ensembl Rapid at the European Bioinformatics Institute ( Table 1; https://rapid.ensembl.org/Spongilla_lacustris_GCA_949361645.1/Info/Index). The resulting annotation includes 43,627 transcribed mRNAs from 30,435 protein-coding and 854 non-coding genes. Alternative annotations can be found here: https://github.com/Aquatic-Symbiosis-Genomics-Project/sponge_annotations.

## Methods

### Sample acquisition and nucleic acid extraction

Tissues from an individual of *Spongilla lacustris* (specimen ID GHC0000126, ToLID odSpoLacu1) was collected from gemmules hatched and grown in the laboratory University of Alberta, Edmonton, Canada (latitude 53.53, longitude –113.53) on 2021-02-06 by using a cell scraper on a Petri dish. The original specimen containing gemmules was collected from O’Connor Lake, Vancouver Island, British Columbia, and was identified by Sally Leys (University of Alberta). The tissues grown in culture were preserved by snap-freezing.

The workflow for high molecular weight (HMW) DNA extraction at the Wellcome Sanger Institute (WSI) includes a sequence of procedures: sample preparation; sample homogenisation, DNA extraction, fragmentation, and clean-up, with all protocols available on protocols.io ( Denton *et al.*, 2023). The odSpoLacu1 sample was weighed and dissected on dry ice ( Jay *et al.*, 2023), and tissue was cryogenically disrupted using the Covaris cryoPREP ^®^ Automated Dry Pulverizer ( Narváez-Gómez *et al.*, 2023). HMW DNA was extracted using the Manual MagAttract v1 protocol ( Strickland *et al.*, 2023b). DNA was sheared into an average fragment size of 12–20 kb in a Megaruptor 3 system ( Todorovic *et al.*, 2023). Sheared DNA was purified by solid-phase reversible immobilisation ( Strickland *et al.*, 2023a), using AMPure PB beads to eliminate shorter fragments and concentrate the DNA. The concentration of the sheared and purified DNA was assessed using a Nanodrop spectrophotometer and Qubit Fluorometer and Qubit dsDNA High Sensitivity Assay kit. Fragment size distribution was evaluated by running the sample on the FemtoPulse system.

RNA was extracted from tissue of odSpoLacu1 in the Tree of Life Laboratory at the WSI using the RNA Extraction: Automated MagMax™ *mir*Vana protocol ( do Amaral *et al.*, 2023). The RNA concentration was assessed using a Nanodrop spectrophotometer and a Qubit Fluorometer using the Qubit RNA Broad-Range Assay kit. Analysis of the integrity of the RNA was done using the Agilent RNA 6000 Pico Kit and Eukaryotic Total RNA assay.

### Sequencing

Pacific Biosciences HiFi circular consensus DNA sequencing libraries were constructed according to the manufacturers’ instructions. DNA sequencing was performed by the Scientific Operations core at the WSI on a Pacific Biosciences SEQUEL II (HiFi)instrument. Hi-C data were also generated from tissue of odSpoLacu1 using the Arima2 kit and sequenced on the Illumina NovaSeq 6000 instrument.

### Genome assembly

The HiFi reads were first assembled using Hifiasm ( Cheng *et al.*, 2021) in primary mode. Haplotypic duplications were identified and removed using purge_dups ( Guan *et al.*, 2020). The Hi-C reads were mapped to the primary contigs using bwa-mem2 ( Vasimuddin *et al.*, 2019). The contigs were further scaffolded using the provided Hi-C data ( Rao *et al.*, 2014) in YaHS ( Zhou *et al.*, 2023) using the --break option for handling potential misassemblies. The scaffolded assemblies were evaluated using Gfastats ( Formenti *et al.*, 2022), BUSCO ( Manni *et al.*, 2021) and MERQURY.FK ( Rhie *et al.*, 2020).

The mitochondrial and chloroplast genomes were assembled using MitoHiFi ( Uliano-Silva *et al.*, 2023), which runs MitoFinder ( Allio *et al.*, 2020) and uses these annotations to select the final contigs and to ensure the general quality of the sequences.

The metagenome assembly was generated using metaMDBG ( Benoit *et al.*, 2024) and binned using MetaBAT2, MaxBin and bin3C. The resulting bin sets of each binning algorithm were optimised and collectively refined using MAGScoT. PROKKA ( Seemann, 2014) was used to identify tRNAs and rRNAs in each bin, CheckM was used to assess bin completeness/contamination, and GTDB-TK ( Chaumeil *et al.*, 2022) was used to taxonomically classify bins. Taxonomic replicate bins were identified using dRep, with default settings (95% ANI threshold). The final bin set was filtered for bacteria and archaea. ‘MAGs’ were categorised as bins with contamination ≤ 5%, identified 5S, 16S, and 23S rRNA genes along with at least 18 unique tRNAs, and either ≥ 90% completeness or ≥ 50% completeness plus fully circularised chromosomes. The remaining bins with ≤ 10% contamination and ≥ 50% completeness and ‘MAGs’ identified as taxonomic replicates were categorised as ‘binned metagenomes’.

### Assembly curation

The assembly was checked for contamination and corrected using the gEVAL system ( Chow *et al.*, 2016) as described previously ( Howe *et al.*, 2021). Manual curation was primarily conducted using PretextView ( Harry, 2022), with additional insights provided by JBrowse2 ( Diesh *et al.*, 2023) and HiGlass ( Kerpedjiev *et al.*, 2018). Any identified contamination, missed joins, and mis-joins were corrected, and duplicate sequences were tagged and removed. The curation process is documented at https://gitlab.com/wtsi-grit/rapid-curation (article in preparation).

### Taxonomic verification

Molecular markers obtained from the assembly were used to reconstruct the phylogenetic position of the sample. For this, a comprehensive dataset of all published freshwater sponge ITS1-5.8S-ITS2 sequences was generated ( *n* = 358) using MAFFT ( Katoh & Standley, 2013), including all 29 specimens currently published as *Spongilla lacustris* from its wide range of its Holarctic distribution. Currently, ITS is regarded as most suitable marker in Spongillida molecular taxonomy ( Erpenbeck *et al.*, 2019). Phylogenetic Analysis with RAxML ( Stamatakis, 2014) recovered the specimen nested among all other samples currently recognised as *S. lacustris* with 97% bootstrap support.

### Evaluation of the final assembly

A Hi-C map for the final host and eukaryote cobiont assemblies were produced using bwa-mem2 ( Vasimuddin *et al.*, 2019) in the Cooler file format ( Abdennur & Mirny, 2020). The MerquryFK tool ( Rhie *et al.*, 2020), run within a Singularity container ( Kurtzer *et al.*, 2017), was used to evaluate *k*-mer completeness and assembly quality for the primary and alternate haplotypes, using the *k*-mer databases ( *k* = 31) computed before genome assembly. The analysis outputs included assembly QV consensus quality values. The genome was also analysed within the BlobToolKit environment ( Challis *et al.*, 2020) and BUSCO scores ( Manni *et al.*, 2021) were calculated.

Table 5 contains a list of relevant software tool versions and sources.

**Table 5.** Software tools: versions and sources.

Software tool	Version	Source
BEDTools	2.30.0	https://github.com/arq5x/bedtools2
bin3C	version 0.3.3	https://github.com/cerebis/bin3C
BlobToolKit	4.2.1	https://github.com/blobtoolkit/blobtoolkit
BUSCO	5.3.2	https://gitlab.com/ezlab/busco
CheckM	1.2.1	https://github.com/Ecogenomics/CheckM
checkM_DB	2015-01-16	
gEVAL	N/A	https://geval.org.uk/
Gfastats	1.3.6	https://github.com/vgl-hub/gfastats
GTDB	214	https://github.com/Ecogenomics/GTDBNCBI
GTDB-TK	2.3.2	https://github.com/Ecogenomics/GTDBTk
Hifiasm	0.16.1	https://github.com/chhylp123/hifiasm
HiGlass	1.11.6	https://github.com/higlass/higlass
MAFFT	7.450	https://mafft.cbrc.jp/alignment/software/
MAGScoT	1.0.0	https://github.com/ikmb/MAGScoT
MaxBin	version 2.7	https://github.com/jtamames/SqueezeMeta/tree/master/bin/MaxBin
Merqury	MerquryFK	https://github.com/thegenemyers/MERQURY.FK
MetaBAT2	version 2.15-15-gd6ea400	https://bitbucket.org/berkeleylab/metabat/src/master/
metaMDBG		https://github.com/GaetanBenoitDev/metaMDBG
MitoHiFi	2	https://github.com/marcelauliano/MitoHiFi
PretextView	0.2	https://github.com/wtsi-hpag/PretextView
PROKKA	1.14.5	https://github.com/vdejager/prokka
purge_dups	1.2.3	https://github.com/dfguan/purge_dups
RAXML	8	https://github.com/amkozlov/raxml-ng
TreeVal	1.0.0	https://github.com/sanger-tol/treeval
YaHS	1.1a.2	https://github.com/c-zhou/yahs

### Genome annotation method

The Ensembl Genebuild pipeline for non-vertebrate species ( Aken *et al.*, 2016) was used to generate annotation for the *Spongilla lacustris* assembly (GCA_949361645.1) in Ensembl Rapid Release at the EBI. Annotation was created primarily through alignment of transcriptomic data to the genome, with gap filling via protein-to-genome alignments of a select set of proteins from UniProt ( UniProt Consortium, 2019).

### Wellcome Sanger Institute – Legal and Governance

The materials that have contributed to this genome note have been supplied by a Tree of Life collaborator. The Wellcome Sanger Institute employs a process whereby due diligence is carried out proportionate to the nature of the materials themselves, and the circumstances under which they have been/are to be collected and provided for use. The purpose of this is to address and mitigate any potential legal and/or ethical implications of receipt and use of the materials as part of the research project, and to ensure that in doing so we align with best practice wherever possible. The overarching areas of consideration are:

•     Ethical review of provenance and sourcing of the material

•     Legality of collection, transfer and use (national and international)

Each transfer of samples is undertaken according to a Research Collaboration Agreement or Material Transfer Agreement entered into by the Tree of Life collaborator, Genome Research Limited (operating as the Wellcome Sanger Institute) and in some circumstances other Tree of Life collaborators.

## Data Availability

European Nucleotide Archive: *Spongilla lacustris*. Accession number PRJEB58940; https://identifiers.org/ena.embl/PRJEB58940. The genome sequence is released openly for reuse. The *Spongilla lacustris* genome sequencing initiative is part of the Aquatics Symbiosis Genomics (ASG) project. All raw sequence data and the assembly have been deposited in INSDC databases. Raw data and assembly accession identifiers are reported in Table 1.

## Author information

Members of the Wellcome Sanger Institute Tree of Life Management, Samples and Laboratory Team are listed here: https://doi.org/10.5281/zenodo.10066175.

Members of the Wellcome Sanger Institute Scientific Operations: Sequencing Operations are listed here: https://doi.org/10.5281/zenodo.10043364.

Members of the Wellcome Sanger Institute Tree of Life Core Informatics team are listed here: https://doi.org/10.5281/zenodo.10066637.

Members of the European Bioinformatics Institute ASG Data Portal team are listed here: https://doi.org//10.5281/zenodo.10076466.

Members of the Wellcome Sanger Institute/Aquatic Symbiosis Genomics Project Leadership are listed here: https://doi.org/10.5281/zenodo.10184833.

## References

[ref-1] Abdennur N Mirny LA : Cooler: scalable storage for Hi-C data and other genomically labeled arrays. *Bioinformatics.* 2020;36(1):311–316. 10.1093/bioinformatics/btz540 31290943 PMC8205516

[ref-2] Adams EDM : Physiology and morphology of epithelia in the freshwater demosponge.Spongilla lacustris (MSc Thesis). University of Alberta, Edmonton,2010.

[ref-57] Aken BL Ayling S Barrell D : The Ensembl gene annotation system. *Database (Oxford).* 2016;2016: baw093. 10.1093/database/baw093 27337980 PMC4919035

[ref-3] Allio R Schomaker-Bastos A Romiguier J : MitoFinder: efficient automated large-scale extraction of mitogenomic data in target enrichment phylogenomics. *Mol Ecol Resour.* 2020;20(4):892–905. 10.1111/1755-0998.13160 32243090 PMC7497042

[ref-4] Benoit G Raguideau S James R : High-quality metagenome assembly from long accurate reads with metaMDBG. *Nat Biotechnol.* 2024;42(9):1378–1383. 10.1038/s41587-023-01983-6 38168989 PMC11392814

[ref-5] Brandt K : Über das Zusammenleben von Algen und Tieren. *Biologisches Centralblatt.* 1881;1:524–527. Reference Source

[ref-6] Brauer EB : Osmoregulation in the fresh water sponge, *Spongilla lacustris*. *J Exp Zool.* 1975;192(2):181–192. 10.1002/jez.1401920208

[ref-7] Brøndsted A Brøndsted HV : The effect of symbiontic zoochlorellae on the germination rate of gemmules of *Spongilla lacustris* (L.). *Dansk Naturhistorisk Forening Videnskabelige Meddelelser.* 1953;115:133–144.

[ref-8] Challis R Richards E Rajan J : BlobToolKit – interactive quality assessment of genome assemblies. *G3 (Bethesda).* 2020;10(4):1361–1374. 10.1534/g3.119.400908 32071071 PMC7144090

[ref-9] Chaumeil PA Mussig AJ Hugenholtz P : GTDB-Tk v2: memory friendly classification with the genome taxonomy database. *Bioinformatics.* 2022;38(23):5315–5316. 10.1093/bioinformatics/btac672 36218463 PMC9710552

[ref-10] Cheng H Concepcion GT Feng X : Haplotype-resolved *de novo* assembly using phased assembly graphs with hifiasm. *Nat Methods.* 2021;18(2):170–175. 10.1038/s41592-020-01056-5 33526886 PMC7961889

[ref-11] Chow W Brugger K Caccamo M : gEVAL — a web-based browser for evaluating genome assemblies. *Bioinformatics.* 2016;32(16):2508–2510. 10.1093/bioinformatics/btw159 27153597 PMC4978925

[ref-12] Denton A Yatsenko H Jay J : Sanger Tree of Life wet laboratory protocol collection V.1. *protocols.io.* 2023. 10.17504/protocols.io.8epv5xxy6g1b/v1

[ref-13] Diesh C Stevens GJ Xie P : JBrowse 2: a modular genome browser with views of synteny and structural variation. *Genome Biol.* 2023;24(1): 74. 10.1186/s13059-023-02914-z 37069644 PMC10108523

[ref-14] do Amaral RJV Denton A Yatsenko H : Sanger Tree of Life RNA extraction: automated MagMax ^TM^ mirVana. *protocols.io.* 2023. 10.17504/protocols.io.6qpvr36n3vmk/v1

[ref-15] Erpenbeck D Steiner M Schuster A : Minimalist barcodes for sponges: a case study classifying African freshwater Spongillida. *Genome.* 2019;62(1):1–10. 10.1139/gen-2018-0098 30557098

[ref-16] Evans KL Montagnes DJS : Freshwater sponge (Porifera: Spongillidae) distribution across a landscape: environmental tolerances, habitats, and morphological variation. *Invertebr Biol.* 2019;138(3):e12258. 10.1111/ivb.12258

[ref-17] Fjerdingstad EJ : The ultrastructure of choanocyte collars in *Spongilla lacustris* (L.). *Zeitschrift Für Zellforschung Und Mikroskopische Anatomie.* 1961;53(5):645–657. 10.1007/BF00339512 13700095

[ref-18] Formenti G Abueg L Brajuka A : Gfastats: conversion, evaluation and manipulation of genome sequences using assembly graphs. *Bioinformatics.* 2022;38(17):4214–4216. 10.1093/bioinformatics/btac460 35799367 PMC9438950

[ref-19] Francis WR Eitel M Vargas S : Mitochondrial genomes of the freshwater sponges *Spongilla lacustris* and *Ephydatia cf. muelleri*. *Mitochondrial DNA B Resour.* 2016;1(1):250–251. 10.1080/23802359.2016.1157771 33473465 PMC7800867

[ref-20] Frost TM : Clearance rate determinations for the freshwater sponge *Spongilla lacustris*: effects of temperature, particle type and concentration, and sponge size. *Archives Fur Hydrobiologie.* 1980;90:330–356.

[ref-21] Frost TM Williamson CE : *In situ* determination of the effect of symbiotic algae on the growth of the fresh water sponge *Spongilla lacustris*. *Ecology.* 1980;61(6):1361–1370. 10.2307/1939045

[ref-22] Gernert C Glöckner FO Krohne G : Microbial diversity of the freshwater sponge *Spongilla lacustris*. *Microb Ecol.* 2005;50(2):206–212. 10.1007/s00248-004-0172-x 16211324

[ref-23] Graffius S Garzón JFG Zehl M : Secondary metabolite production potential in a microbiome of the freshwater sponge *Spongilla lacustris*. *Microbiol Spectr.* 2023;11(2): e0435322. 10.1128/spectrum.04353-22 36728429 PMC10100984

[ref-24] Guan D McCarthy SA Wood J : Identifying and removing haplotypic duplication in primary genome assemblies. *Bioinformatics.* 2020;36(9):2896–2898. 10.1093/bioinformatics/btaa025 31971576 PMC7203741

[ref-25] Harrison FW Rosenberg EM Davis DA : Correlation of cyclic GMP and cyclic AMP immunofluorescence with cytochemical patterns during dormancy release and development from gemmules in *Spongilla lacustris* L. (Porifera: Spongillidae). *J Morphol.* 1981;167(1):53–63. 10.1002/jmor.1051670106 30114857

[ref-26] Harry E : PretextView (Paired REad TEXTure Viewer): a desktop application for viewing pretext contact maps.2022. Reference Source

[ref-27] Howe K Chow W Collins J : Significantly improving the quality of genome assemblies through curation. *GigaScience.* 2021;10(1): giaa153. 10.1093/gigascience/giaa153 33420778 PMC7794651

[ref-28] Huss VAR Dörr R Grossmann U : Deoxyribonucleic acid reassociation in the taxonomy of the genus *Chlorella*. *Arch Microbiol.* 1986;145(4):329–333. 10.1007/BF00470866

[ref-29] Imsiecke G : Ingestion, digestion, and egestion in *Spongilla lacustris.* (Porifera, Spongillidae) after pulse feeding with *Chlamydomonas reinhardtii* (Volvocales). *Zoomorphology.* 1993;113(4):233–244. 10.1007/BF00403314

[ref-30] Jay J Yatsenko H Narváez-Gómez JP : Sanger Tree of Life sample preparation: triage and dissection. *protocols.io.* 2023. 10.17504/protocols.io.x54v9prmqg3e/v1

[ref-31] Katoh K Standley DM : MAFFT multiple sequence alignment software version 7: improvements in performance and usability. *Mol Biol Evol.* 2013;30(4):772–80. 10.1093/molbev/mst010 23329690 PMC3603318

[ref-32] Kerpedjiev P Abdennur N Lekschas F : HiGlass: web-based visual exploration and analysis of genome interaction maps. *Genome Biol.* 2018;19(1): 125. 10.1186/s13059-018-1486-1 30143029 PMC6109259

[ref-33] Kurtzer GM Sochat V Bauer MW : Singularity: scientific containers for mobility of compute. *PLoS One.* 2017;12(5): e0177459. 10.1371/journal.pone.0177459 28494014 PMC5426675

[ref-34] Lewin RA : Kultivo de zoochlorella apartigita el spongo. *Sci Rev Int Sci Assoc Esperantista.* 1966;17:33–36.

[ref-35] Manconi R Pronzato R : Global diversity of sponges (Porifera: Spongillina) in freshwater.In: Balian, E. V., Lévêque, C., Segers, H., and Martens, K. (eds.) *Freshwater Animal Diversity Assessment.*Netherlands, Dordrecht: Springer,2008;27–33. 10.1007/s10750-007-9000-x

[ref-36] Manni M Berkeley MR Seppey M : BUSCO update: novel and streamlined workflows along with broader and deeper phylogenetic coverage for scoring of eukaryotic, prokaryotic, and viral genomes. *Mol Biol Evol.* 2021;38(10):4647–4654. 10.1093/molbev/msab199 34320186 PMC8476166

[ref-37] Muscatine L Karakashian SJ Karakashian MW : Soluble extracellular products of algae symbiotic with a ciliate, a sponge and a mutant hydra. *Comp Biochem Physiol.* 1967;20(1):1–12. 10.1016/0010-406X(67)90720-7

[ref-38] Musser JM Schippers KJ Nickel M : Profiling cellular diversity in sponges informs animal cell type and nervous system evolution. *Science.* 2021;374(6568):717–723. 10.1126/science.abj2949 34735222 PMC9233960

[ref-39] Narváez-Gómez JP Mbye H Oatley G : Sanger Tree of Life sample homogenisation: Covaris cryoPREP ^®^ Automated Dry Pulverizer V.1. *protocols.io.* 2023. 10.17504/protocols.io.eq2lyjp5qlx9/v1

[ref-40] Pröschold T Darienko T : *Choricystis* and *Lewiniosphaera* gen. nov. (Trebouxiophyceae Chlorophyta), two different green algal endosymbionts in freshwater sponges. *Symbiosis.* 2020;82(3):175–188. 10.1007/s13199-020-00711-x 33328698 PMC7725700

[ref-41] Rao SSP Huntley MH Durand NC : A 3D map of the human genome at kilobase resolution reveals principles of chromatin looping. *Cell.* 2014;159(7):1665–1680. 10.1016/j.cell.2014.11.021 25497547 PMC5635824

[ref-42] Rhie A Walenz BP Koren S : Merqury: reference-free quality, completeness, and phasing assessment for genome assemblies. *Genome Biol.* 2020;21(1): 245. 10.1186/s13059-020-02134-9 32928274 PMC7488777

[ref-43] Saller U : Oogenesis and larval development of *Ephydatia fluviatilis* (Porifera, Spongillidae). *Zoomorphology.* 1988;108(1):23–28. 10.1007/BF00312211

[ref-44] Saller U : Symbiosis of *Spongilla lacustris* (Spongillidae) and Green Algae. Algae uptake, distribution and final whereabouts.In: Reitner, J. and Keupp, H. (eds.) *Fossil and Recent sponges.*Berlin: Springer-Verlag,1991;299–305. 10.1007/978-3-642-75656-6_23

[ref-45] Saller U Weissenfels N : The development of *Spongilla lacustris* from the oocyte to the free larva (Porifera, Spongillidae). *Zoomorphology.* 1985;105(6):367–374. 10.1007/BF00312280

[ref-46] Seemann T : Prokka: rapid prokaryotic genome annotation. *Bioinformatics.* 2014;30(14):2068–2069. 10.1093/bioinformatics/btu153 24642063

[ref-47] Stamatakis A : RAxML version 8: a tool for phylogenetic analysis and post-analysis of large phylogenies. *Bioinformatics.* 2014;30(9):1312–1313. 10.1093/bioinformatics/btu033 24451623 PMC3998144

[ref-48] Strickland M Cornwell C Howard C : Sanger Tree of Life fragmented DNA clean up: manual SPRI. *protocols.io.* 2023a. 10.17504/protocols.io.kxygx3y1dg8j/v1

[ref-49] Strickland M Moll R Cornwell C : Sanger Tree of Life HMW DNA extraction: manual MagAttract. *protocols.io.* 2023b. 10.17504/protocols.io.6qpvr33novmk/v1

[ref-50] Todorovic M Sampaio F Howard C : Sanger Tree of Life HMW DNA fragmentation: diagenode Megaruptor ^®^3 for PacBio HiFi. *protocols.io.* 2023. 10.17504/protocols.io.8epv5x2zjg1b/v1

[ref-51] Uliano-Silva M Ferreira JGRN Krasheninnikova K : MitoHiFi: a python pipeline for mitochondrial genome assembly from PacBio high fidelity reads. *BMC Bioinformatics.* 2023;24(1): 288. 10.1186/s12859-023-05385-y 37464285 PMC10354987

[ref-58] UniProt Consortium: UniProt: a worldwide hub of protein knowledge. *Nucleic Acids Res.* 2019;47(D1):D506–D515. 10.1093/nar/gky1049 30395287 PMC6323992

[ref-52] Vasimuddin M Misra S Li H : Efficient architecture-aware acceleration of BWA-MEM for multicore systems.In: *2019 IEEE International Parallel and Distributed Processing Symposium (IPDPS).*IEEE,2019;314–324. 10.1109/IPDPS.2019.00041

[ref-53] Wachtmann D Stockem W Weissenfels N : Cytoskeletal organization and cell organelle transport in basal epithelial cells of the freshwater sponge *Spongilla lacustris*. *Cell Tissue Res.* 1990;261(1):145–154. 10.1007/BF00329447

[ref-54] Weissenfels N Wachtmann D Stockem W : The role of microtubules for the movement of mitochondria in pinacocytes of fresh-water sponges (Spongillidae, Porifera). *Eur J Cell Biol.* 1990;52(2):310–4. 2081532

[ref-55] Williamson CE : An ultrastructural investigation of algal symbiosis in white and green *Spongilla lacustris* (L.) (Porifera: Spongillidae). *Trans Am Microsc Soc.* 1979;98(1):59–77. 10.2307/3225940

[ref-56] Zhou C McCarthy SA Durbin R : YaHS: yet another Hi-C scaffolding tool. *Bioinformatics.* 2023;39(1): btac808. 10.1093/bioinformatics/btac808 36525368 PMC9848053

