# Supplementary material for: The chromosome-level genome sequences of the freshwater sponge, Spongilla lacustris (Linnaeus, 1759) and the chlorophyte cobiont Choricystis sp., and the associated microbial metagenome sequences
Source: Wellcome Open Res. 2025 Apr 25;10:222. [Version 1] doi: 10.12688/wellcomeopenres.23988.1 (PMC12141999; doi:10.12688/wellcomeopenres.23988.1)
Supplement: Peer review history

# Reviewer report: Reviewer response for version 1

**DOI:** 10.21956/wellcomeopenres.26464.r123946

**Published:** 2025-06-05

**Authors:** Matsapume Detcharoen

**Affiliations:** 1 Prince of Songkla University, Hat Yai, Thailand

This study by Leys and the team reports high quality genome sequences of *Spongilla lacustris *and its green algal cobiont, *Choricystis* sp. The sponge’s genome spans 248.7 Mb, with 23 chromosomal pseudomolecules and the algal cobiont’s genome is 14.6 Mb.

 One important aspect of the work is its integrative scope. Not only does the paper provide the host sponge genome, but it also reports a genome for the intracellular green algae and recovers three high quality bacterial genomes from the metagenomic data. This broad perspective reflects the complexity of the sponge holobiont.

 I have a couple of minor comments, the interactive metagenome blob plot is currently not working and the BUSCO completeness for the *Spongilla *genome is moderate, which may reflect biological complexity or technical challenges.

Are sufficient details of methods and materials provided to allow replication by others?

Yes

Is the rationale for creating the dataset(s) clearly described?

Yes

Are the datasets clearly presented in a useable and accessible format?

Yes

Are the protocols appropriate and is the work technically sound?

Yes

Reviewer Expertise:

host-symbiont interaction, genomics

I confirm that I have read this submission and believe that I have an appropriate level of expertise to confirm that it is of an acceptable scientific standard.

# Reviewer report: Reviewer response for version 1

**DOI:** 10.21956/wellcomeopenres.26464.r122911

**Published:** 2025-06-02

**Authors:** Laura Steindler

**Affiliations:** 1 University of Haifa, Haifa, Haifa, Israel

The article provides a well-structured and informative background on the sponge species *Spongilla lacustris*, which was sequenced in this study. The background covers key aspects of the species, including its geographic distribution, macro- and micro-morphology, reproductive biology, and symbiotic relationships. *S. lacustris* represents a particularly compelling model for studying sponge biology and host-microbe interactions, as it can be grown under laboratory conditions. Notably, the sequenced specimen was derived from gemmules that were hatched and reared in the lab.

 An important contribution of this work is the simultaneous sequencing, assembly, and annotation of the genome of *Choricystis*, a chlorophyte cobiont harbored by *S. lacustris*. Previous studies have shown that these algal cells reside intracellularly, particularly within choanocytes and archaeocytes, adding another layer of interest to this symbiotic system.

 In addition, the authors report the assembly of three bacterial genomes, affiliated with the families Flavobacteriaceae and Oxalobacteraceae. However, as noted in the background, earlier research has characterized the mesohyl of *S. lacustris* as largely free of resident bacterial symbionts, with bacteria only observed as digested material in archaeocytes. Therefore, in future studies, it will be important to clarify whether the assembled bacterial genomes represent true symbionts or originate from transient, free-living bacteria ingested as part of the sponge’s diet.

 Overall, the article is clearly written and the genomic data provided will serve as a valuable resource for future studies on freshwater sponges and their symbiotic interactions.

**Minor corrections**: The names *Hydra* and *Paramecium* should be italicized in the Background section.

 The link to the interactive version of figure 6 is not working.

Are sufficient details of methods and materials provided to allow replication by others?

Yes

Is the rationale for creating the dataset(s) clearly described?

Yes

Are the datasets clearly presented in a useable and accessible format?

Yes

Are the protocols appropriate and is the work technically sound?

Yes

Reviewer Expertise:

Sponge symbiosis, marine microbiology

I confirm that I have read this submission and believe that I have an appropriate level of expertise to confirm that it is of an acceptable scientific standard.
